# The Bicycle and Tricycle for Physicians

**Published:** 1883-07

**Authors:** 


### THE BICYCLE AND TRICYCLE FOR PHYSICIANS.

A new era is rapidly dawning upon the world's locomotion. Little did the most visionary dreamer imagine that in the child's plaything, the English velocipede, there was the germ of a machine developing destined to revolutionize the world's minor locomotion. But so it is, and the successful general introduction of the bicycle awaits only the smoothing and consolidating of the dirt roads and streets of our country and cities. Macadamize is the motto of the times, and there is an urgent reason for this, irrespective of the use of the bicycle.

Some fifteen years ago the cumbrous velocipede was introduced from the smooth turnpikes of England into the United States, but it was soon discovered in both countries that a reinvention of the velocipede was needed quite as much as macadamization of highways before the velocipede would be anything more than a laborious, locomotive toy for children. Dr. G. E. Corbin, of Michigan, thus describes the subsequent improvement of the velocipede into the graceful bicycle of to-day:

"From this crude beginning has been constructed the bicycle so arranged as to sustain the weight of the rider but slightly posterior to the axle of the front wheel, which possesses a radius but little less than the length of the leg of the rider. In this erect position most of the weight of the body can easily be transmitted to the cranks for the purpose of propulsion. If all the weight were sustained by the feet, and thereby transmitted to the cranks, it would require no more muscular exertion than would walking with the feet equally wide apart, a distance of ten or twelve inches. The expenditure of such an amount of power, however, is never required for its propulsion, except in the act of climbing hills, or wading through sand or mud.

On smooth, hard, level roads, two thirds of the weight of the rider may rest upon the saddle, and the other third alternating from one pedal to the other will rotate the wheel with ease and speed. One third the weight of the body thrown upon one foot while describing one-half of the circumference of a circle ten or twelve inches in diameter will convey the rider to the distance of one-half the circumference of the larger wheel. On a 54 inch wheel, the average size ridden, this slight effort will convey the rider a distance of about seven feet, In other words, thrusting the foot through a space of from ten to fourteen inches, with a power sufficient to sustain only one-third the weight of the body, will convey the rider a greater distance than will three full steps of a pedestrian while sustaining the entire weight of the body.

This theoretic view is confirmed by actual experience. On good roads I can ride my bicycle ten miles in less time and with less exertion than are required for me to walk three miles. That ambitious young riders cover from fifty to one hundred miles daily for many days in succession, is undeniable. That a light, graceful vehicle, possessing such advantages, can and will become a useful aid to physicians as a means of locomotion, can scarcely admit of a doubt.

This in no way implies that human muscle is to be substituted for "horseflesh."

The exercise of a short ride in an easy carriage is only passive. Notwithstanding the long daily rides of many physicians are laborious, the weariness is produced by a long continued series of concussions and oscillations that do not inflate the lungs, expand the chest and exercise the muscles to the best advantage.

In this condition, both pleasure and rest may be secured by a thirty or sixty minutes' ride upon the bicycle, and it may just as well be taken in visiting patients as otherwise. Physicians, dentists and others who are confined closely in offices, school rooms, counting rooms, etc., will find the bicycle a very fascinating and beneficial means of exercise. Its skillful use can be speedily and easily acquired.

Its use requires just enough attention to divert the mind from the excessive strain of business, and this is no small point gained.

With most persons it can be sufficiently used without interfering with regular hours of business.

Its use inflates the lungs, expands the chest, exercises, develops and strengthens every muscle in the body.

To those who (if any such there be) imagine that bicycle riding does not fully comport with the dignity of their stations, I would suggest that whatever in the way of garb, vehicle, instruments or appliances is most appropriate for the occasion; whatever will most effectually and easily accomplish any worthy object sought, is entirely justifiable. and can by no possibility detract from merited dignity.

Concerning the bicycle I do not speak from theory alone. I was half a century old before I ever mounted one, and am now riding mine for the third season, and with much individual benefit. I have riden my bicycle with ease and pleasure twenty-five miles in an afternoon, over our country roads, which are not the best, making several professional calls during the time.

To my professional brethren I would say, test the wheel for yourselves.

